# Taxonomic efforts in deep sea hydrothermal vents are approaching total coverage

**DOI:** 10.1098/rspb.2026.2250

**Published:** 2026-11-11

**Authors:** Julia D. Sigwart, Chong Chen

**Affiliations:** ^1^ Senckenberg – Leibniz Institution for Biodiversity and Earth System Research, Senckenberg Institute and Natural History Museum Frankfurt, Frankfurt 60325, Germany; ^2^ Institute for Ecology, Diversity and Evolution, Goethe University Frankfurt, Frankfurt 60438, Germany; ^3^ X-STAR, Japan Agency for Marine-Earth Science and Technology, Yokosuka 237-0061, Japan

**Keywords:** species richness, taxonomy, deep sea, rarefaction, predictive model, biodiversity

## Abstract

Despite centuries of taxonomic research, an estimated approximately 90% of marine invertebrate species remain undescribed. Taxonomy must become more efficient for policy and conservation decisions; however, we know little about how taxonomic practices have changed over recent decades. We used ChatGPT 4.0 to extract metadata from the original descriptions of all 588 species endemic to deep sea hydrothermal vents, and examined temporal trends in species discovery, authorship, institutional and national representation, and description length. A typical species description has about 600 words, is in English, lacks a diagnosis and systematic revision, has two authors usually from the same country and is published around 6 years after discovery. Description length decreases with author experience and international collaborations are dramatically more concise. The global species accumulation curve for vents is plateauing, with the East Pacific Rise and Northern Mid-Atlantic Ridge already approaching an asymptote. The median projected global richness is 788 (maximum 1060) endemic species. Although considerable biodiversity remains undescribed, three-quarters of global vent-endemic species have been described and complete description can be achieved within the current generation of taxonomists. This metadata-extraction approach reveals generalizable patterns in taxonomy and offers a scalable way to quantify effort and predict completion timelines for other poorly known taxa.

## 1. Introduction

Despite centuries of taxonomic effort, many clades and regional faunas remain incompletely described [1]. The taxonomic impediment is perhaps felt most acutely in marine systems, which contain the highest phylogenetic diversity as the majority of animal phyla do not occur in terrestrial environments [2,3]. Previously published overviews including animals, plants and microbes estimated that over 90% of marine species remained undescribed [4,5]. Globally, new marine species are named at a rate of about 2000 per year [2], but this is inadequate relative to the over 1 million undescribed species [4]. Multiple recent efforts to increase the pace of discovery and especially of description are particularly focused on the urgent conservation needs for marine systems [6–8]. Understanding the historical pace as well as trends in lengths and details of species descriptions across taxa is key to predicting the speed at which taxonomy can progress in the foreseeable future. A complete biodiversity and taxonomic baseline is the fundamental framework for fields of research, such as systematics, conservation and ecological inference. Furthermore, scientific names are also the basis for policy-related and legal actions for species protection, such as assessing species for the International Union for Conservation of Nature (IUCN) Red List and the establishment of protected areas [8]. Hydrothermal vents are a useful testbed, with a clearly contained fauna and strong conservation relevance that can offer transferable insights to other ecosystems.

Projecting the total species richness and the time needed to describe them requires examining not only how many species have been described, or those that were discovered but remain unnamed, but also examining how species descriptions are produced and published [2]. Species descriptions offer rich metadata, as they are structured records of how taxonomy is practised. Each description captures multiple attributes of the process, including technical, systematic and sociological information: authorship, institutional affiliation, length and format of the account, and the timing of the research and publication process. These features provide a window into the organization of taxonomic work, allowing quantitative assessments of how descriptions are produced, by whom and under what constraints. Analysing such metadata therefore offers a means to examine the structure, efficiency and equity of taxonomic knowledge production, in addition to information about quantifying biodiversity.

Shifting patterns in publication dynamics reflect broader changes in society or discipline-specific practices. A general long-term trend for increasing collaboration in authorship of scientific papers is not equally reflected in all disciplines [9]. Taxonomic papers generally still have fewer authors [10,11], although the last few years have seen multi-author compilations dedicated to accelerating species description [12]. Trends in species and paper authors indicate how participation in taxonomy is distributed and how it is changing [2,13]. At the same time, variation in description length and publication tempo may signal differences in descriptive standards, editorial expectations or the complexity of the taxa being treated. In marine species, most differences are anecdotally understood as differences in practice among phyla. For example, descriptions of arthropods, such as isopods, rely on time-consuming line drawings and list extensive details of each body part (e.g. [14]), while those of molluscs, such as gastropods, would typically rather use photographs and electron micrographs (e.g. [15]). Patterns can be quantified directly from species description metadata, enabling comparisons across time, taxonomic groups and regions, and providing insight into how the practice of taxonomy itself is evolving.

Species descriptions accumulate through time in a manner that can be used to assess the completeness of taxonomic knowledge. Species accumulation curves provide a framework to evaluate whether discovery is approaching saturation within a given ecosystem or region and to estimate the likely total diversity based on observed trends [13,16]. These projections are crucial for setting research priorities, estimating total diversity and anticipating diminishing returns as the pace of discovery begins to slow down.

Here, we leverage species description metadata for the global deep sea hydrothermal vent ecosystem to examine how the process of taxonomy itself is changing, and what it tells us about the likely completeness of species discovery in this system. Hydrothermal vents offer a unique opportunity to assess a phylogenetically diverse community with clearly demarcated temporal and geographic boundaries. Vents were first discovered on the Galápagos Rift in 1977 [17], followed by major discoveries on the East Pacific Rise (EPR) in 1979 [18], the Northern Mid-Atlantic Ridge (NMAR) in 1985 [19] and the Indian Ocean in 2000 [20]. These deep sea geysers host a unique ecosystem supported by bacterial primary production through chemosynthesis, using reducing chemicals in the geofluids, such as hydrogen sulfide and methane; many animals live in symbiosis with such bacteria or use them as the only source of food [21,22]. The concentration of hydrogen sulfide and other toxic substances, such as heavy metals, in the vent fluids means only specially adapted species can live within the vent ecosystem. These factors lead to a fauna where the majority of species found at vents are endemic and do not occur anywhere else [23,24].

Over 600 hydrothermal vents have been discovered to date worldwide (666 confirmed or inferred as being active) [25,26], together hosting about 600 vent-endemic species [23] which are distributed as segregated, regionally distinct faunas [27–29]. It is possible to identify the complete set of all global taxonomic descriptions of species that are endemic to vents, and to make comparisons in practice across different phyla. A comprehensive taxonomic corpus for this whole ecosystem could be compiled with high confidence. Analysis of these taxonomic works provides a case study to examine several fundamental questions pertinent to all biodiversity research: how can metadata inform systematic research agendas? What are the social and structural dynamics shaping species description? And finally, can we predict the true global species richness of a major ecosystem?

## 2. Methods

### (a) Species list compilation

A list of benthic vent-endemic metazoan species was initially compiled based on the sFDvent database [23], incorporating all animal phyla excluding Chordata and Nematoda. We expanded the list with further species described after that publication, assembling a list of all hydrothermal vent species described until the end of 2024 (supplementary material). We then manually checked the species list and removed all taxa that have also been found at non-vent habitats after description or those suspected to be non-endemic owing to sampling locations from vent peripheral habitats. We then retrieved the citation and a PDF file of the publication for every original taxonomic paper with the species descriptions, a corpus of 335 articles and book chapters. Many publications include multiple species and sometimes also treated non-vent-endemic taxa. Further analyses used a dataset compiled for each species, including a unique identifier for the relevant description paper.

### (b) Metadata extraction

We used the large language model ChatGPT (GPT-4.0; OpenAI, www.chatgpt.com) to automate the extraction of metadata from each publication. The analyses were completed using a legacy version of GPT4.0 during May 2025. This included: (i) publication-level metadata, including the publication journal and language, the authors and the countries of their listed affiliations, and (ii) species-level metadata, including the species authority (which may differ from the authors of the paper), count of figures included in the description, information on type specimens and the species etymology (table 1). The metadata extraction was performed by uploading one PDF and a README file with the instructions (supplementary material) in a new chat window within the ChatGPT online interface. Analysing a single file at a time ensured that the model did not confuse or combine information from multiple papers. The routine for each paper produced a line of tab-delimited metadata that was compiled into a single table for all species.

**Table 1.** Field definitions for the metadata extracted from original species descriptions and used in further comparisons.

paper-level metadata	
paper_id	file name of the PDF
journal_name	full name of the journal
year_of_publication	year the paper was published
language_of_paper	language the paper is written in
number_of_authors	total number of paper authors
author_countries_list	list of all countries represented by author affiliations
number_of_author_countries	count of distinct countries in author affiliations
first_author_country	country of affiliation of the first author
number_of_species_in_paper	total number of species and subspecies described in this paper
systematic_revision_present	true if the paper includes a genus- or family-level revision

species-level metadata	
species_name	full binomial
species_authority	taxonomic authority as given in paper
species_author_list	list of names of species authors
number_of_species_authors	count of species authors
species_author_countries_list	countries of affiliation for species authors
number_of_species_author_countries	distinct countries for species authors
first_species_author_country	country of the first-named species author
figures_cited_in_description	count of figures cited
figure_parts_count	count of all numbered illustrations (e.g. figure 1a–c = 3 parts)
year_of_first_record	year the species was first collected (not necessarily described)
year_record_flag_manual_check	true if year is from a citation (not specimen data) and needs curation.
type_locality_latitude / type_locality_longitude	type locality location
region_in_words	geographic region in plain language
minimum_depth_m / maximum_depth_m	depth range of specimens in metres
etymology_type	etymology representing one of: person, place, morphology, other or ambiguous
etymology_text	explanation of the name (verbatim or summarized)
holotype_repository	institution holding the holotype specimen
paratype_repositories	list of institutions with paratypes
paratype_repositories_count	number of unique institutions with paratypes
collecting_vessels	research vessels or submersibles involved in collecting the type material

For each species, we recorded the word count of the formal taxonomic diagnosis (if present) and description. For the word count analysis, we decided *a priori* that the ‘description’ includes all text and subheadings that are directly related to the description of the species and the type material. Ecological notes that directly concern the single species of interest are included when they occur in the same (sub)section of the paper as the description. Taxonomic remarks, comparative remarks or phylogenetic discussions are not included. Clearly separated subsections for ‘material examined’ or specimen measurements are not included, as these may comprise museum catalogue numbers or abbreviations that are not equivalent to natural language word counts (e.g. body length given as ‘BL 10 mm’).

Extracted data were extensively spot-checked to validate the accuracy of the approach. Fields for country names, institution names and vessel names were further curated to ensure consistent formatting. The combined data from manual checks (description length) and semi-automated extraction (publication data and species description metadata) were compiled into a single dataset for statistical analyses (supplementary material). The list of species retrieved by the metadata extraction process was validated with currently accepted names via the World Register of Marine Species (WoRMS) Taxon Match tool (https://www.marinespecies.org/aphia.php?p=match), and then the list of accepted names of retrieved species was compared against the original target list.

### (c) Data analyses

The main curated dataset was used to analyse trends over time and comparative trends in different phyla for a number of variables (table 1). In analyses of temporal trends, we excluded two vent-endemic species described from serendipitous sampling events much earlier than the discovery of vent ecosystems themselves: the amphipod *Autonoe longicornis* (Chevreux, 1909) and the bivalve *Wareniconcha lepta* (Dall, 1896). Statistical analyses were completed in R v. 4.5.1 [30]; the full documentation to produce analyses, figures and tables is provided in the supplementary material.

One hypothesis is that individual authors become more concise with experience. In order to test these patterns of behaviour among authors and the correlation between career experience and other output variables such as description length, we isolated a set of author names that are believed to represent unique individuals, and extracted the full career output of these names in marine invertebrate taxonomy, based on all species recorded in the WoRMS database (https://www.marinespecies.org/). We extracted all species authorities from 1950 to 2024 from a full download of WoRMS (May 2025). Unlike botanical authorities [31], zoological taxonomy does not implement a disambiguated representation of individual authors. We first excluded commonly occurring surnames (e.g. Smith and Zhang), and then filtered the total list to extract species described by author names matching those who have described vent-endemic species. Names with an apparent career longer than 40 years (first and last publication per name) were checked manually to exclude any representing a combination of work from multiple scientists with the same surname. This process produced a list of unique authors, the years of their taxonomic publications, and the number of vent species and total species they described in successive years (supplementary material). This was used to test the potential correlation between total career experience and word count of vent-endemic species descriptions.

Furthermore, we completed a rarefaction analysis in order to estimate the global vent species richness (including projected species that currently remain undescribed). Each species was assigned to one of three target biogeographic regions discovered between 1977 and 2000 (northern EPR, NMAR and Indian Ocean) based on their type localities and latitude–longitude boundaries (supplementary material); species described from outside these boundaries were grouped in the ‘other’ region. For each region and year, we quantified annual and cumulative species discovery rates (discovery here is defined as the first collection of the species, based on year of first record as reported in the taxonomic description) and description rates based on year of publication, and calculated the lag between the first record and the formal description, when available. Temporal trends in discovery and description rates were summarized using descriptive statistics and by comparing early versus recent thirds of each region's time series. We fitted multiple candidate accumulation models (linear, logarithmic, Michaelis–Menten, asymptotic exponential, Gompertz and logistic) to regional cumulative richness curves and selected the best-fitting model by using the Akaike Information Criterion. Asymptotic richness estimates and their standard errors were extracted from the best-performing model for each region. We further quantified the levels of uncertainty using 1000-iteration nonparametric bootstrap resampling of regional time series, refitting the best model to each bootstrap sample to derive confidence intervals for total richness and the proportion of species already described.

## 3. Results

Our final vent-endemic species dataset included 588 species meeting our criteria (i.e. vent endemic, described before 2025, currently valid), from an original set of 598 species that were used to compile the literature corpus. Some species were removed during final manual data curation (*n* = 23). ChatGPT correctly identified 13 additional vent-endemic taxa within the literature corpus that had not been included in the original target list. There were also species retrieved in the metadata extraction that were excluded from further analysis based on evidence that they were not vent endemic, but most of these could not have been deduced from the description paper alone (i.e. they were found in other ecosystems after the description, or they became synonymized), so this cannot be considered an error by ChatGPT. Errors by ChatGPT occurred with 47 species in two main types. Firstly, ChatGPT retrieved data for 10 species that were not newly described in the paper; generally, these were redescriptions. Secondly, the first pass of the extraction routine also skipped 37 species that should have been retrieved when they were in larger papers containing many (more than 10) species; when the instructions were modified to request species in batches of five instead of all species included, ChatGPT returned the correct expected output for all taxa in these long papers. In total, the target-species error rate was up to approximately 8% on the first pass by ChatGPT. Although extensive cross-checking and manual curation of the data were required, the use of automation through a large language model did result in an enhanced dataset and net time saving.

### (a) Trends in vent taxonomic descriptions

Based on metadata trends, a typical species description is around 600 words long, written in English, does not include a diagnosis or any systematic revision, is authored by two people usually from the same country and is published around 6 years after the species was first discovered (table 2; supplementary material, figure S1). The length of the description text does vary in small but statistically significant ways among the three dominant phyla (Annelida, Arthropoda and Mollusca; figure 1). The mean word count is highest among arthropods and lowest in molluscs. The mean description word count is not changing over time in the total dataset, but decreasing in annelids (figure 1). About 40% of species descriptions include a diagnosis section, although over time descriptions have increasingly included a diagnosis (table 2). The lag from discovery to description is increasing over time; the median lag is the highest in molluscs (table 2) and echinoderms (supplementary material, figure S1).

**Table 2.** Summary statistics for key metrics and trends obtained from vent-endemic species descriptions. For categorical variables, trends reflect the frequency of the dominant value over time. Data for most variables are segmented and compared between the dominant phyla (Mollusca, Arthropoda and Annelida); the phyla with the lowest and highest values are shown, indicating either the mean value within the phylum or the low and high frequency of the dominant value for categorical variables.

metric	mean / dominant value	total trend over time	comparison among phyla	phylum with lowest value	phylum with highest value
species described per year	11.7 ± 9 species per year	no change (*p* = 0.409)	significant difference (*p* = 0.0289)	Annelida (average = 2.4)	Arthropoda (average = 4.6)
**continuous variables**					
description word count	672 ± 22 words	no change (*p* = 0.998)	significant difference (*p* < 0.001)	Mollusca (467)	Arthropoda (906)
lag	6.4 ± 0.2 years	increasing (*p* < 0.001)	significant difference (*p* = 0.0002)	Arthropoda (5.6)	Mollusca (7.6)
number of paper authors	2.2 ± 0.08 authors	increasing (*p* < 0.001)	significant difference (*p* = 0.0135)	Arthropoda (1.9)	Annelida (2.5)
number of author countries	1.5 ± 0.03 countries	increasing (*p* < 0.001)	significant difference (*p* = 0.0001)	Arthropoda (1.3)	Mollusca (1.6)
number of techniques	2.1 ± 0.04 techniques	increasing (*p* < 0.001)	significant difference (*p* < 0.001)	Arthropoda (1.7)	Mollusca (2.5)
collecting depth	2057 ± 33 m	decreasing (*p* = 0.0001)	no difference (*p* = 0.41)	Mollusca (1996 m)	Annelida (2137 m)
**categorical variables**					
language of paper	English (96.6%)	increasing (*p* = 0.001)	no difference (*p* = 0.42)	Annelida (95%)	Mollusca (97.7%)
etymology inspiration	morphology (41.0%)	decreasing (*p* < 0.001)	significant difference (*p* = 0.0012)	Annelida (27.3%)	Mollusca (47.9%)
diagnosis	included 39.6%	increasing (*p* < 0.001)	significant difference (*p* = 0.0085)	Annelida (26.4%)	Mollusca (42.9%)
systematic revision	included 39.6%	decreasing (*p* < 0.001)	no difference (*p* = 0.14)	Arthropoda (34.9%)	Mollusca (43.4%)
molecular data	included 23.5%	increasing (*p* < 0.001)	significant difference (*p* = 0.0027)	Arthropoda (16.4%)	Annelida (30.6%)

**Figure 1.**
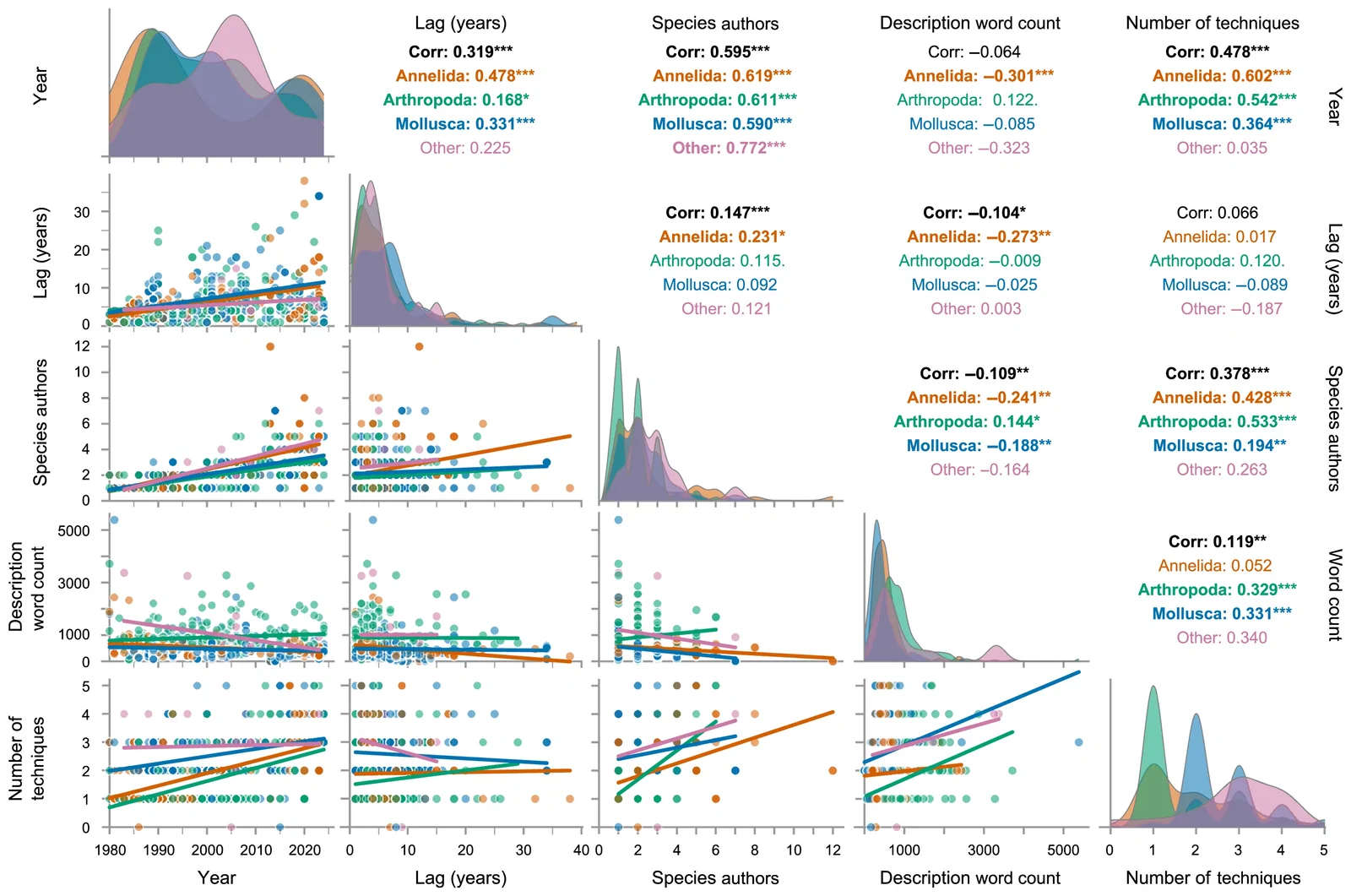
Matrix scatterplot of variables describing species descriptions, showing the trends segmented by phyla (Annelida, Arthropoda, Mollusca and all others). The plots compare year of publication, lag (years elapsed) from first reported discovery to published description, number of authors per species, the word count of single species descriptions, and the number of distinct laboratory techniques included in the description text or illustrations. Upper-right panels report Pearson correlation coefficients overall and by phylum (*p < 0.05, **p < 0.01, ***p < 0.001); lower-left panels show linear fits. Panels in the middle diagonal show the density distribution of each variable by phylum.

Around 26% of vent species are named after people, and the use of honorifics has shifted, as earlier species names clearly favoured morphological features over eponyms (supplementary material, figure S2). The technical data used varies by organismal group (figure 1; supplementary material, figure S2). The number of techniques used increased over time in all three main phyla, with Arthropoda descriptions typically using the fewest techniques (table 2). *In situ* photographs are increasingly accessible for deep sea research but are still used in less than half of recent species descriptions. Molecular work was effectively unavailable in the 1980s and is now used in about three-quarters of descriptions; meanwhile, the use of drawings has declined from nearly all species descriptions to around 25% or less (supplementary material, figure S2).

Taxonomy is becoming more collaborative, with increasing numbers of authors per species and per paper, and international collaboration has increased markedly compared with earlier decades (figures 1 and 2A). The length of a species description becomes more concise with author experience (figure 2B). More strikingly, international collaboration is correlated with shorter word counts (figure 2C). Species descriptions typically cite type material deposited in multiple countries, but the number of countries receiving type material for each species is apparently not increasing over time (figure 2A).

**Figure 2.**
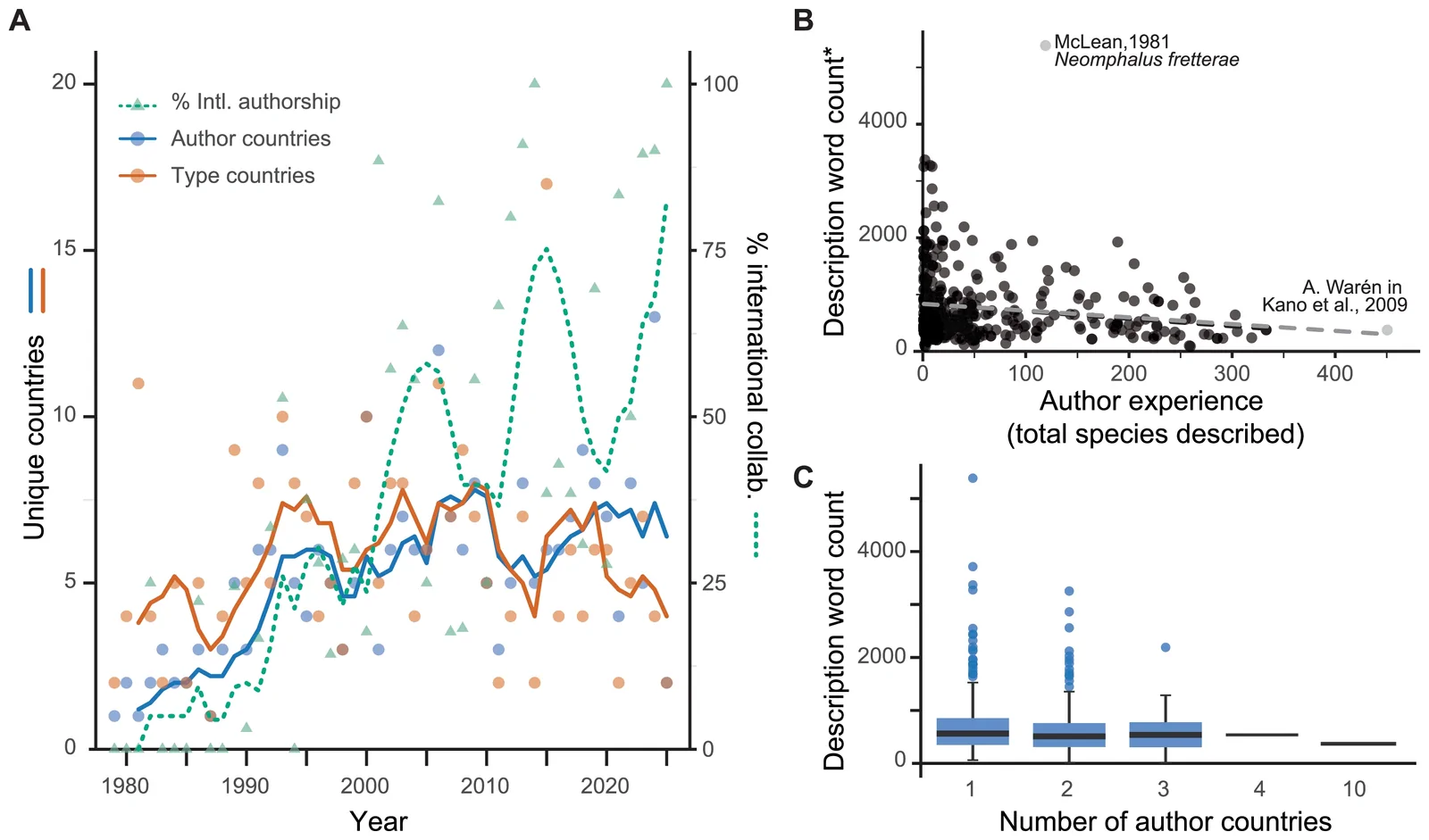
The influence of authors' total experience in vent-endemic species descriptions. (A) Scatterplot contrasting the number of countries (left axis) counted among the species authors (blue) and the number of countries receiving type material from each newly described species (orange). The percentage of species including international collaboration (two or more countries) (green triangles) is shown on the right axis. Trend lines indicate a 5 year moving average. (B) Description word count compared with the total number of species described by its contributing authors; this plot is based on a subset of species descriptions that can be confidently ascribed to a unique author, and species with multiple authors are represented multiple times in the plot. (C) Box plot of median and inner quartile values for the description word count for species with single, 2, 3, 4 or 10 authors per species.

### (b) Predicting the total vent-endemic species diversity

Rarefaction analyses used species (*n* = 585) with clearly georeferenced type locality data. Cumulative species discovery curves show that the global species richness of vent-endemic species is approaching an asymptote, and the number of undiscovered species can be predicted with confidence (figure 3). Approximately 12 species are described per year on average and are not increasing (table 2). Values in past years were certainly higher (figure 1), but this represents a past rapid increase after the initial discovery of vent systems that has now stabilized to a more steady rate of description.

**Figure 3.**
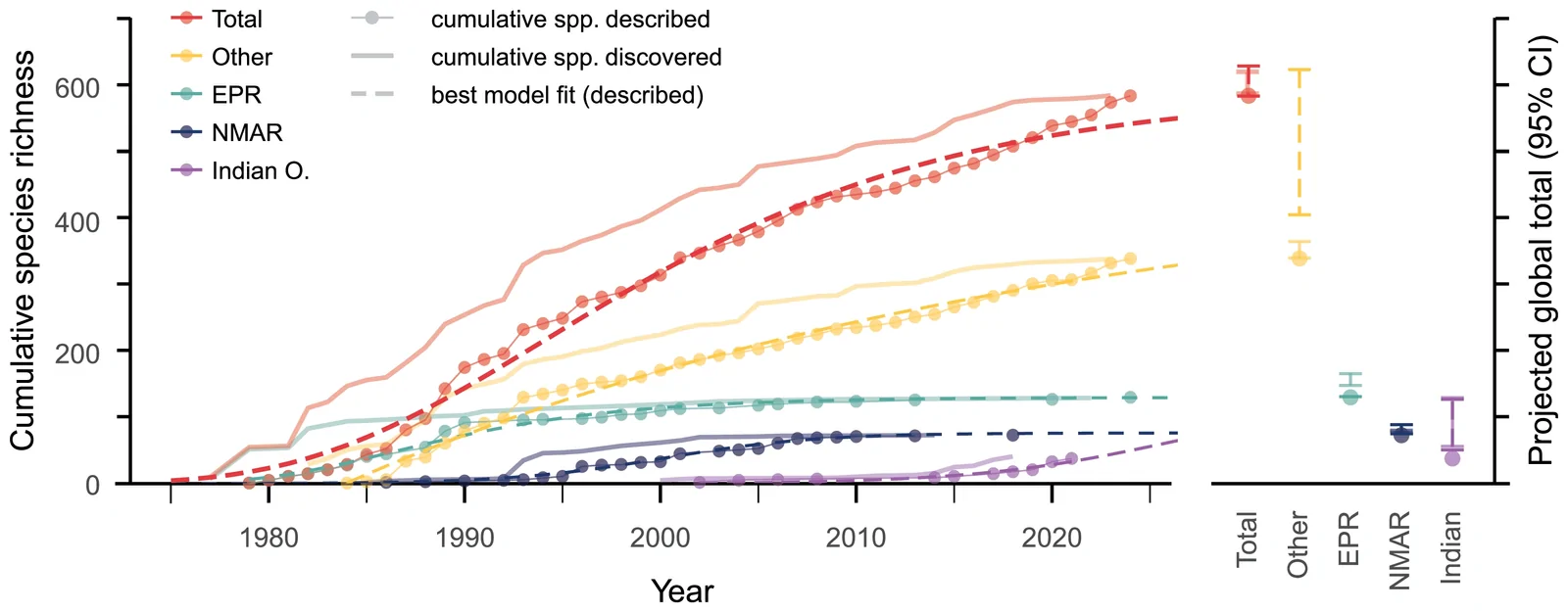
Rarefaction projection of the total species richness of vent-endemic benthic species. The data are geographically segmented based on the type locality of each species into separate faunas in the EPR, NMAR, Indian Ocean and all other vent areas, as well as the total. Dots indicate the cumulative number of species described, which lags the cumulative trend in species discovered (line), but these two lines should intersect as the fauna approaches total description. The dashed line shows the growth model fitted to the species description rate. The panel to the right shows 95% confidence intervals for model projections of the total fauna based on both description (dashed line) and discovery rates (solid line).

Regional predictions based on discovery or description rates are consistent for the northern EPR and NMAR where the species accumulation curves are plateauing. In the northern EPR, the maximum predicted number of species is 157, compared with 130 currently recognized species; in the NMAR, the maximum prediction is 83 compared with 74 known species. There is more uncertainty for the predicted number of species in the Indian Ocean, where a plateau is not in sight and reliable projection is not achieved (42–408 spp. in different approaches). For the ‘other’ geographic regions outside our focal areas, the uncertainty is also relatively high, but the projection based on the year of publication (‘described’ range: 446–521 species) is much higher than the better-resolved projection based on the year of first occurrence (‘discovered’ confidence interval range: 343–419 spp.; figure 3). Using the sum of regional projections to incorporate additional uncertainty, the maximum predicted global number of vent-endemic species is 1060 and is likely lower (minimum estimate is 607 species; supplementary material, table S1). The median projected value across all models is 788 vent-endemic species globally.

## 4. Discussion

The patterns in our dataset reflect both epistemological saturation for the hydrothermal vent ecosystem and shifting dynamics in how taxonomic knowledge is produced. The global hydrothermal vent system overall is approaching a point where most species that can be found have already been described (figure 3), meaning that discovery rates naturally begin to slow down, and the remaining species are rarer, harder to find or harder to identify or delimit from other similar species. The global plateauing is mainly owing to the fauna of the well-studied EPR and NMAR systems being effectively completely described, while more discoveries are still expected for other systems, for example in the Indian Ocean. Global rarity and inaccessibility are inherent characteristics of deep sea species, and this common ground underlines the robust nature of the globally saturating species accumulation curve at vents. The saturation is not an artefact of intermittent pulses of discovery, as the rates of deep sea exploration at hydrothermal vents are currently accelerating as an effect of exploratory cruises related to deep sea mining [32–34]. The trends over time in our dataset also reveal changes in the process of doing taxonomy, unrelated to the underlying biodiversity, in authorship patterns (more collaboration), publication practices, methods (e.g. molecular data), and institutional and geographic participation.

### (a) Temporal patterns in taxonomic descriptions

Scientific papers in general are becoming more concise over time, with shorter text length but longer reference lists [35]. This trend is interpreted as an effect of publication- and citation-based metrics for evaluating academic performance, which favour both many publications and many citations [36]. We did not measure the total word count of the papers in this dataset or the number of references included; we could not automate word counting tasks with accuracy, because the nature of ChatGPT and similar large language models operate by learning patterns in texts, and their outputs are fundamentally generative estimates [37]. Nonetheless, our description word count information shows no overall change over time (table 2). The lack of change in description length may reflect the detachment of taxonomic work from broader competitive processes in academia, and the long-standing problem that taxonomic papers are under-cited [38,39]. One striking realization is that most species descriptions do not include a diagnosis, a pattern largely congruent across phyla (table 2)—although there is an increase in descriptions with diagnosis over time—showing that there are stylistic changes within the modern discipline.

Although we show a trend of more experienced authors becoming more concise (figure 2B), vent species offer some biological surprises that confound this pattern. The level of anatomical adaptation to the extreme environment of hydrothermal vents has led to many anatomical and phylogenetic novelties, such as the giant tubeworm *Riftia pachyptila*, initially thought to be a new phylum, and the scaly-foot gastropod *Chrysomallon squamiferum* [40] with iron-infused scales [41,42]. In some cases, the species description and anatomical descriptions are published as separate studies (e.g. [40,43,44]). The first publication of the gastropod *Neomphalus fretterae* [45] is an extraordinarily long description of a single species (5385 words for the description section; figure 2B) that reflects the importance of this taxon at the moment of its discovery [45,46]. *Neomphalus* was immediately recognized as representing a new taxonomic order, and therefore the description contains a phenomenally detailed anatomical analysis that served to put this species in the context of global gastropod diversity.

Scientific papers in general are also becoming more collaborative [47], but taxonomy as a discipline lags behind the global trend [11]. Although there is a statistically significant increase in both paper authors and species authors, most descriptions still have three or fewer authors. Having few authors is linked to the old tradition of the discipline in keeping species authors to three or fewer to avoid lengthy citations of taxonomic authorities [48]. One outlier to this is the recently launched publication series *Ocean Species Discoveries*, which publishes collaborative compilations of concise marine invertebrate descriptions [12]. The first paper in the *Ocean Species Discoveries* series included two vent-endemic molluscs and other papers in this series post-date our cut-off at the end of 2024. The *Ocean Species Discoveries* dramatically extends the maximum number of paper authors connected to mollusc species to 25 co-authors, although the respective species had only three species authors from one country (Japan) [12]. This is a sign of a trend in embracing collaborations, which may lead to rapid shifts in the taxonomic landscape in the next decade [8].

International co-authorship in species descriptions has continued to increase over time (figure 2A), and unexpectedly we found that an increasing number of author countries correlates strongly with shorter species descriptions (figure 2C). Another surprising pattern was the overall increase in the use of names honouring people (supplementary material, figure S2B), which is in conflict with recent discourse among taxonomists on the use of eponyms as being potentially offensive [49]. We speculate that the increased use of honorifics is owing to vent taxonomy being a relatively young field; the first generation of vent taxonomists has only become sufficiently well-established in the last couple of decades to attract species dedications by the new generation [50]. The initial surge of species descriptions following the discovery of vents clearly favoured morphology-based names, perhaps owing to the unusual anatomical features of many vent taxa, as mentioned above. We also note that although the vast majority of species descriptions included the named deployable platform (submersible or remotely operated vehicle) that collected the specimens (lacking in 37 descriptions), many did not specify the research vessel (lacking in 151 descriptions). The lack of ship information in the papers made it challenging to analyse the patterns in national efforts, and we recommend that vent taxonomists take care in noting down key collecting data, such as ship and submersible names, when describing species.

New technologies that provide additional lines of evidence for species delimitation or diagnosis have been integrated into vent taxonomy as they become available. Overall, the use of drawings is decreasing while photographs, electron microscopy and molecular data are increasing (supplementary material, figure S2D). Scanning electron microscopy was relatively inaccessible at the start of our dataset 40–50 years ago [51], and is now taken for granted as a standard part of species descriptions in many taxa. Species descriptions also increasingly incorporate molecular data, but around 80% of new and known marine invertebrate species in most clades still do not include any DNA data [2]. The percentage of vent-endemic species descriptions that include molecular data (18%) is similar to the overall number for marine invertebrates, though the rate is much higher when later follow-up studies on these species are included. We note that the increasing availability and the need to use different techniques in descriptions over time [11] are factors contributing to the increasing number of authors (figure 1).

### (b) Differences among taxonomic groups

How much is enough for a species description? This depends on the expectation of the community and the level of clarity about the useful diagnostic characters in the group being described. Taxonomic practitioners have recognized anecdotal differences in species descriptions for different major clades, such as the description of crustaceans, which are typically lengthy, as they have been standardized to include every single character in every appendage regardless of their usefulness in diagnosis [52,53], whereas those of shelled molluscs have been traditionally short and often based on distinguishing shell characters only [54]. It has been unclear, however, to what extent these differences genuinely separate the taxonomic descriptions in various groups or if they are exaggerated idiosyncrasies.

The overarching patterns observed from our vent dataset challenge such assumptions about strong disciplinary differences among phyla, as the key aspects of the ‘typical’ vent species description (Results) apply more or less to all major groups and the differences among groups are mostly small even when statistically significant (table 2). For example, the average description length of arthropoda (906) is indeed longer than mollusca (567), but both are well within the same order of magnitude. The mean number of techniques used is higher in Mollusca (2.5) than in Arthropoda (1.7), but in reality, this means both use around two techniques per species. There is generally a high level of consistency in taxonomic practice and the patterns observed across time, which is transferable across phyla (figure 1; table 2).

We did find some clear differences in the preference of techniques among phyla that characterize how species in each phylum are described (supplementary material, figure S2C). Arthropod descriptions rely heavily on drawings, while only about one-third include photographs. Mollusc descriptions show the opposite pattern, where they almost always include photographs and scanning electron micrographs and only less than one-third include drawings. Annelid descriptions are more diverse, though most descriptions use drawings and about half include photographs and scanning electron micrographs. Cnidarian taxonomists almost always include photographs, histology and *in situ* photographs but rarely use drawings.

Taxonomists describing deep sea biodiversity are required to recognize, delimit and interpret species in systems where baseline data are sparse and access is limited [55]. This specialist knowledge is particularly critical in the emerging context of deep sea mining, where environmental impact assessments, biodiversity baselines and monitoring frameworks depend on the accurate identification of species [56]. Without taxonomic expertise, species may remain unrecognized or conflated, undermining assessments of distribution, connectivity and extinction risk [57]. As a result, taxonomists are central to translating biological complexity into operational knowledge for management decisions in these ecosystems [8]. Our meta-analyses reveal some different expectations and disciplinary norms across major taxonomic groups, which are helpful to support inclusive participation.

### (c) Predicting the true global species richness of a major ecosystem

Our rarefaction analyses reveal a plateauing cumulative species richness curve for the global hydrothermal vent ecosystem (figure 3), allowing us to predict the true global species diversity of vent-endemic fauna as being around 788 species (median of all prediction models, range: 607–1060 species; supplementary material, table S1). The fauna of non-endemic species connected to vents is larger and is not included here; changes in the understanding of vent endemism were not captured in our analyses, so no effect of species is removed from the total as they are discovered in other ecosystems. With 588 recognized, strictly vent-endemic species being already described until the end of 2024 (supplementary material), we have already described the majority of all vent-endemic benthic animals globally.

The number of species described lags species discovered by about 5–10 years, as can be seen from the horizontal separation of the different aspects of the plot (figure 3). As these discovered species represent the years reported in the published species description, these data do not capture unpublished information or preliminary reports of additional known but undescribed species (e.g. [58]). These two lines should intersect as the fauna approaches total description, as we observe for the two best-studied systems EPR and NMAR. Likewise, the confidence intervals of the models from both discovery and description rates should give the same or similar range when the discovery rate is truly approaching saturation (supplementary material, table S1). For both EPR (130 described species and projected range 130–157 species) and NMAR (74 described species and projected range 73–83 species), the taxonomic work is already nearly complete.

Hydrothermal vents in the Indian Ocean were discovered about 20 years later than those in EPR and NMAR [20], with new vent sites still being frequently discovered [59,60]. Compared with EPR and NMAR, the Indian Ocean cumulative richness curve has not plateaued (figure 3), and there is still a large fraction of this fauna yet to be described, as noted in biogeographic analyses and faunal lists [29,58]. The lack of plateauing leads to much larger uncertainty in the number of total taxa present in the Indian Ocean and ‘other’ regions outside these three target biogeographic regions, and divergence in the confidence intervals of the discovery- and description-based models. This is a reflection of recent new discoveries of additional hydrothermal vent systems in previously unexplored ocean basins (e.g. Woodlark Basin in the southwestern Pacific [61]) and regions (e.g. Arctic Ocean; [62]) that remain under-described. A large portion of the undescribed vent-endemic fauna likely constitutes those inhabiting the understudied vent periphery where specialists of inactive chimneys occur [63], although such inactive vents are now also targeted by deep sea mining and therefore their exploration is expected to increase in the coming years with impacts to that broader fauna [64].

## 5. Conclusions

Complete description of biodiversity, of ecosystems and taxonomic groups and eventually our planet, is important as a fundamental framework upon which all later researchers depend. Policy and conservation tools, such as the IUCN Red List, also depend on species having scientific names [8]. Globally well-studied groups, such as birds and large mammals [65,66], are effectively completely described. The last discovery of an ungulate was the saola in 1992 [67], the first in over 50 years at that time, and in 2026 researchers described the first new cat species in over a century [68]. For well-studied clades, the complete and robust knowledge of global species diversity positions these groups to be used as supposedly definitive models for investigations into deeper and finer-scale questions in biology, such as trait evolution or biogeography [69].

Where the fauna or the group is poorly known, it becomes important to capture all possible characters that may become useful later and these observations may produce longer descriptions. But long descriptions can also reflect taxonomists' excitement about the extreme novelty of a particular species. Having a robustly described fauna makes it faster and more efficient to describe new species, yet also more exciting in terms of scientific impact when new species are found; this is the case in birds or mammals, and some invertebrate groups where there is an active, cohesive taxonomic community. Poorly known groups have long seemed too far behind for this kind of completeness to feel attainable, but that gap is largely unmeasured and without quantitative benchmarks of how fast description happens and what drives it. The structured, comparable approach we present here is a case study that makes that gap measurable, and shows it can be closed.

The goal of complete description is now in reach for the global hydrothermal vent ecosystem. Even with more taxonomic work to be expected, especially in the Indian Ocean, knowing that about three-quarters of the global vent-endemic biodiversity has already been described means we can be confident that the complete description of the global hydrothermal vent fauna will be achieved within the working lifetime of the current generation of deep sea taxonomists. We are looking forward to that day, moving us beyond the discovery-exploration phase at hydrothermal vents and into using this unique fauna as a model system for deep questions in evolutionary biology and ecology. Vents will likely become the first deep sea ecosystem, if not the first marine system, to achieve near-complete faunal descriptions. Although vents are unusual in that the harsh environmental conditions have led to a relatively low species richness [24,27], the fact that it is possible to complete faunal descriptions for an inaccessible deep sea ecosystem is a positive outlook for other deep sea ecosystems such as cold seeps, seamounts or manganese nodule fields [70]. The metadata-extraction approach applied here offers a practical tool to quantify current description effort, flag where taxonomic capacity is lagging and project realistic completion timelines, helping prioritize resources before habitats are lost. Accelerating the rate of descriptions at vent systems remains urgent owing to the imminent threat of deep sea mining [8,71], but our results project a bright future where we can give names to all vent animals, especially before those living in regions targeted by deep sea mining face extinction from anthropogenic impacts.

## Supplementary Material



## Data Availability

Original data files are available at Zenodo [72]. Supplementary material is available online [73].
