# Supplementary material for: Taxonomic efforts in deep sea hydrothermal vents are approaching total coverage
Source: Proc Biol Sci. 2026 Nov 11;293(2082):20262250. doi: 10.1098/rspb.2026.2250 (PMC13751117; doi:10.1098/rspb.2026.2250)
Supplement: Supplementary file 1 [file rspb.2026.2250.zip › rspb.2026.2250/rspb20262250_si_001.zip!/vent lag supplement/Supplementary figures.pdf]

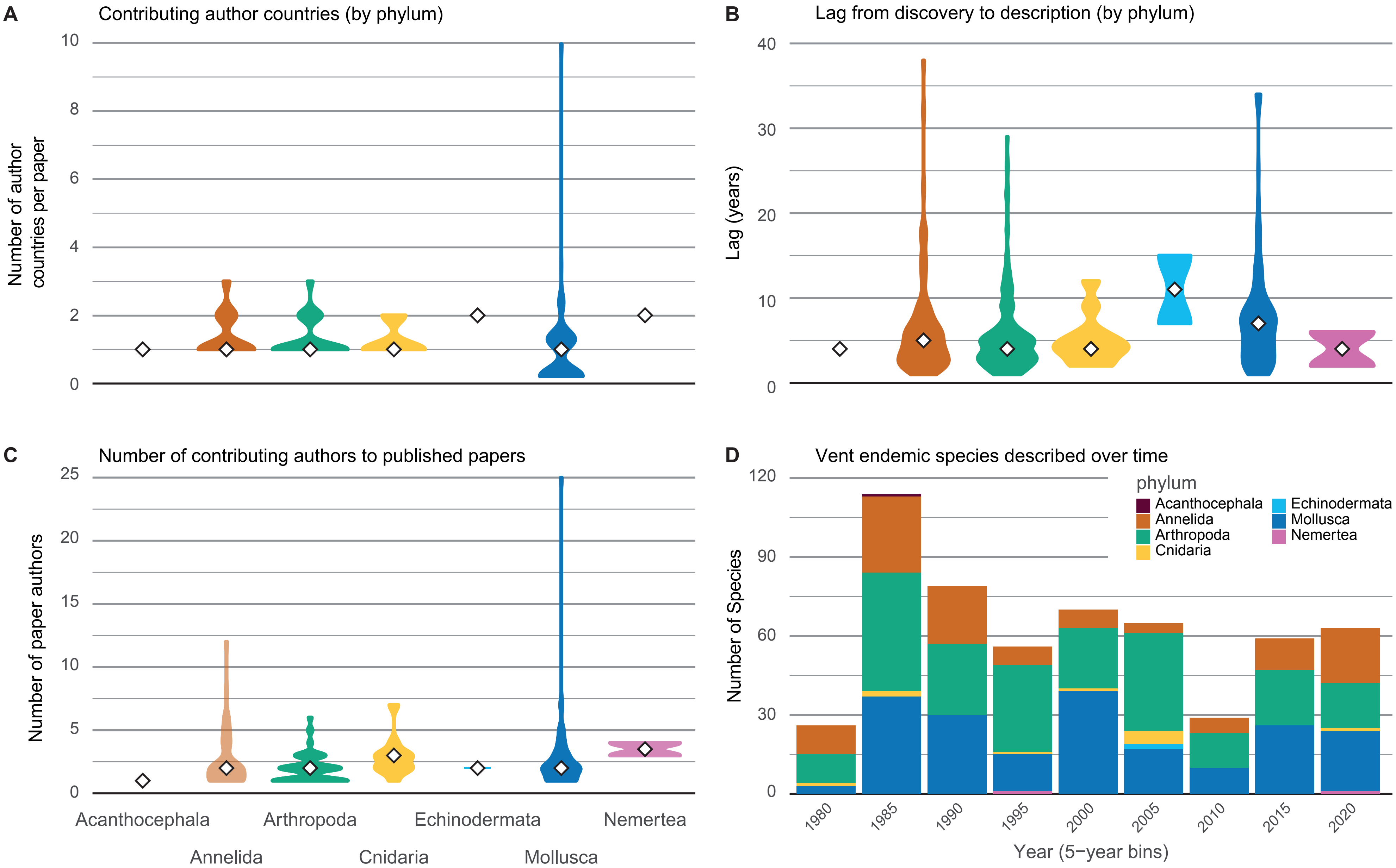

Supplementary Figure 1 (data from 1975-2024)

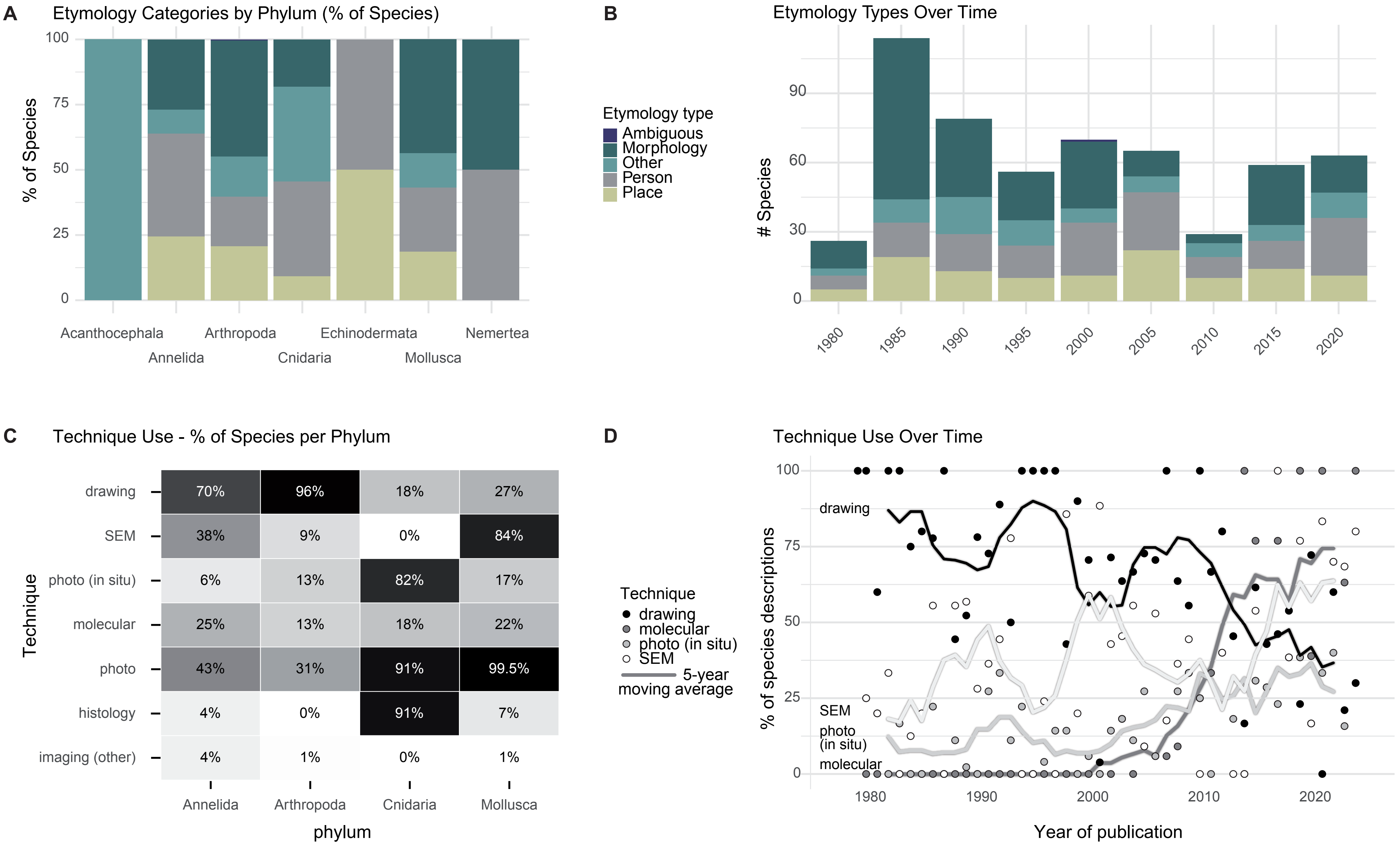

Supplementary Figure 2 (data from 1975-2024)
